# Supplementary material for: Rapid Detection of Pityophthorus juglandis (Blackman) (Coleoptera, Curculionidae) with the Loop-Mediated Isothermal Amplification (LAMP) Method
Source: Plants (Basel). 2021 May 22;10(6):1048. doi: 10.3390/plants10061048 (PMC8224600; doi:10.3390/plants10061048)
Supplement: Supplementary file 1 [file plants-10-01048-s001.zip › plants-1183868-supplementary.pdf]

plants

MDPI

Article

Rapid Detection of *Pityophthorus Juglandis* (Blackman) (Coleoptera, Curculionidae) with the Loop-Mediated Isothermal Amplification (LAMP) Method

Domenico Rizzo [1], Salvatore Moricca [2,*], Matteo Bracalini [2], Alessandra Benigno [2], Umberto Bernardo [3], Nicola Luchi [4], Daniele Da Lio [5], Francesco Nugnes [3], Giovanni Cappellini [1], Chiara Salemi [5], Santa Olga Cacciola [6] and Tiziana Panzavolta [2]

Supplementary:

Table S1. Tamp means ±SD for adult and frass samples of *P. juglandis*.

N°	Description	Tamp Means	SD	N°	Description	Tamp Means	SD	Mean Melting Temperatures (°C)	SD
1	*P. juglandis*_adult	6.06	0.02	1	*P. juglandis*_frass	12.26	0.70	90.75	0.35
2	*P. juglandis*_adult	6.06	0.04	2	*P. juglandis*_frass	12.90	1.20	91.00	0.00
3	*P. juglandis*_adult	6.04	0.07	3	*P. juglandis*_frass	13.30	1.69	91.00	0.00
4	*P. juglandis*_adult	6.23	0.16	4	*P. juglandis*_frass	12.63	2.60	90.50	0.00
5	*P. juglandis*_adult	6.18	0.08	5	*P. juglandis*_frass	13.23	1.64	90.50	0.00
6	*P. juglandis*_adult	6.22	0.05	6	*P. juglandis*_frass	12.29	0.74	91.00	0.00
7	*P. juglandis*_adult	6.17	0.11	7	*P. juglandis*_frass	12.88	1.08	90.75	0.35
8	*P. juglandis*_adult	6.24	0.08	8	*P. juglandis*_frass	13.31	1.69	90.75	0.35
9	*P. juglandis*_adult	6.38	0.02	9	*P. juglandis*_frass	13.33	1.66	91.00	0.00
10	*P. juglandis*_adult	6.32	0.04	10	*P. juglandis*_frass	12.54	1.22	91.00	0.00
11	*P. juglandis*_adult	6.44	0.03	11	*P. juglandis*_frass	12.35	0.82	90.50	0.00
12	*P. juglandis*_adult	6.17	0.08	12	*P. juglandis*_frass	12.31	0.77	90.50	0.00
13	*P. juglandis*_adult	6.08	0.06	13	*P. juglandis*_frass	12.88	0.90	91.00	0.00
14	*P. juglandis*_adult	6.10	0.09	14	*P. juglandis*_frass	13.32	1.01	90.75	0.35
15	*P. juglandis*_adult	6.42	0.12	15	*P. juglandis*_frass	13.34	0.96	91.00	0.00
16	*P. juglandis*_adult	6.48	0.02	16	*P. juglandis*_frass	12.54	0.92	91.00	0.00
17	*P. juglandis*_adult	6.58	0.05	17	*P. juglandis*_frass	12.41	0.87	90.75	0.35
18	*P. juglandis*_adult	6.24	0.04	18	*P. juglandis*_frass	12.94	0.83	91.00	0.00
19	*P. juglandis*_adult	6.42	0.02	19	*P. juglandis*_frass	13.32	0.79	91.00	0.00
20	*P. juglandis*_adult	6.43	0.14	20	*P. juglandis*_frass	14.24	0.75	90.5	0.00
21	*P. juglandis*_adult	6.45	0.02	21	*P. juglandis*_frass	13.20	0.71	90.5	0.00
22	*P. juglandis*_adult	6.47	0.15	22	*P. juglandis*_frass	13.23	0.67	91.00	0.00
23	*P. juglandis*_adult	6.49	0.16	23	*P. juglandis*_frass	13.25	0.63	90.75	0.35
24	*P. juglandis*_adult	6.51	0.08	24	*P. juglandis*_frass	13.28	0.59	91.00	0.00

Table S2. Tamp means ±SD for the samples included in the blind panel.

N°	Description	Tamp Means	SD	Mean Melting Temperatures (°C)	SD
1	*P. juglandis*_adult	8.78	0.32	91.00	0
2	*P. juglandis*_adult	8.78	0.26	91.00	0
3	*P. juglandis*_adult	9.10	0.21	90.50	0
4	*P. juglandis*_adult	8.24	0.06	91.00	0
5	*P. juglandis*_adult	8.78	0.08	91.00	0
6	*P. juglandis*_adult	8.74	0.05	90.50	0
7	*P. juglandis*_frass	13.20	0.02	90.75	0.35
8	*P. juglandis*_frass	12.95	0.18	90.50	0
9	*P. juglandis*_frass	12.89	0.12	91.00	0
10	*P. juglandis*_frass	12.85	0.48	91.00	0
11	*P. juglandis*_frass	13.20	0.08	90.50	0
12	*P. juglandis*_frass	12.98	0.24	90.50	0
13	*P. juglandis*_frass	12.89	0.60	91.00	0
14	*P. juglandis*_frass	12.90	0.20	90.75	0.35
15	*Pityophthorus pubescens* (Marsham)_adult	n/a	0.00	n/a	0.00
16	*Ips sexdentatus* (Börner)_adult	n/a	0.00	n/a	0.00
17	*Ips typographus* (Linnaeus)_adult	n/a	0.00	n/a	0.00
18	*Orthotomicus erosus* (Wollaston)_adult	n/a	0.00	n/a	0.00
19	*Hylurgus ligniperda* (Fabricius)_adult	n/a	0.00	n/a	0.00
20	*Tomicus destruens* (Wollaston)_adult	n/a	0.00	n/a	0.00
21	*Xyleborinus saxesenii* (Ratzeburg)_adult	n/a	0.00	n/a	0.00
22	*Xuleborus dispar* (Fabricius)_adult	n/a	0.00	n/a	0.00
23	*Xyleborus monographus* (Fabricius)_adult	n/a	0.00	n/a	0.00
24	*Xylosandrus compactus* (Eichhoff)_adult	n/a	0.00	n/a	0.00
25	*Xylosandrus germanus* (Blandford)_adult	n/a	0.00	n/a	0.00
26	*Lepturges confluens* (Haldeman)_adult	n/a	0.00	n/a	0.00
27	*Xylosandrus crassiuculus* (Motschulsky)_adult	n/a	0.00	n/a	0.00
28	*Zeuzera pyrina* (Linnaeus)_larva	n/a	0.00	n/a	0.00
29	*Xylosandrus compactus* (Eichhoff)_frass	n/a	0.00	n/a	0.00
30	*Xylosandrus compactus* (Eichhoff)_frass	n/a	0.00	n/a	0.00
31	*Xylosandrus compactus* (Eichhoff)_frass	n/a	0.00	n/a	0.00
32	*Xylosandrus compactus* (Eichhoff)_frass	n/a	0.00	n/a	0.00
